# Investigation of the Optimal Duration and Modality for Postoperative Surveillance of Intraductal Papillary Mucinous Neoplasm (IPMN): A Single-Center Retrospective Study

**DOI:** 10.3390/diagnostics16050803

**Published:** 2026-03-08

**Authors:** Akane Ozawa, Atsushi Nara, Kota Yokoyama, Junichi Tsuchiya, Daisuke Ban, Ukihide Tateishi

**Affiliations:** 1 Department of Diagnostic Radiology, Institute of Science Tokyo, Tokyo 113-8510, Japan; 2 Department of Hepatobiliary and Pancreatic Surgery, Institute of Science Tokyo, Tokyo 113-8510, Japan

**Keywords:** intraductal papillary mucinous neoplasm (IPMN), postoperative surveillance, contrast-enhanced computed tomography (CT)

## Abstract

**Background/Objectives**: Although multiple guidelines exist for the management of intraductal papillary mucinous neoplasms (IPMN), the duration and modality of postoperative surveillance remain inconsistent. We aimed to retrospectively review medical images of patients with IPMN after surgery and to investigate the optimal surveillance duration and modality. **Methods**: In this study, we included 191 patients with IPMN who underwent surgery at a single institution between January 2006 and May 2024. Patients were followed from the postoperative period until July 2025. Image interpretation reports written by diagnostic radiologists were examined to determine the time to recurrence detection and the imaging modality used. **Results**: Sixteen patients (8.3%) were eligible during the observation period. Seven patients experienced intrapancreatic recurrence, and ten patients experienced extrapancreatic recurrence (one patient was included in both categories). The mean time to identification of intrapancreatic lesions was 63.9 months; five of seven cases were detected using contrast-enhanced computed tomography (CT). The mean time to identification of extrapancreatic lesions was 12.0 months, which was significantly shorter than that for intrapancreatic lesions (*p* = 0.005). Eight of ten extrapancreatic recurrences were detected using contrast-enhanced CT. **Conclusions**: Extrapancreatic lesions appeared earlier after IPMN surgery than intrapancreatic lesions. Contrast-enhanced CT was the most commonly used modality for detecting recurrent lesions, suggesting its usefulness in postoperative surveillance.

## 1. Introduction

Intraductal papillary mucinous neoplasm (IPMN) is a mucin-producing epithelial tumor that originates from the pancreatic ductal system. It is characterized by the formation of papillary epithelial proliferations, the presence of multilocular cystic lesions, and dilation of the main pancreatic duct or its branches. These pathological and radiological features distinguish IPMN from other cystic lesions of the pancreas.

Since the 1980s, the reported prevalence of IPMN has steadily increased. This rise is thought to be largely attributable to significant advances in diagnostic imaging modalities, such as computed tomography (CT) and magnetic resonance imaging (MRI), as well as growing clinical awareness of IPMN and its potential for malignant transformation into pancreatic cancer [1,2]. In support of this trend, a large retrospective cohort study analyzing CT images obtained between 2000 and 2015 demonstrated that IPMN was present in 10.9% of individuals aged 50 years or older, indicating that IPMN is relatively common in the aging population [3].

IPMN represents a wide pathological spectrum, ranging from benign lesions to overtly malignant disease. Histologically, IPMN is classified into low-grade dysplasia (LGD), high-grade dysplasia (HGD), and invasive carcinoma (IC). When preoperative clinical evaluation and imaging findings suggest the presence of HGD or IC, surgical resection is generally recommended to prevent disease progression. However, due to improvements in early detection, a substantial proportion of IPMN cases are now diagnosed and surgically treated while still at a noninvasive stage.

In addition to histopathological classification, IPMN is also categorized according to its morphological features into branch duct (BD) type and main duct (MD) type, based on the site and pattern of ductal involvement. Lesions that demonstrate characteristics of both categories are further classified as mixed-type IPMNs. These morphological subtypes are clinically important because they are associated with markedly different risks of malignant transformation. Malignancy rates are relatively low in BD-IPMNs (4–46%), and these lesions are generally associated with a lower risk of progression, allowing for careful imaging surveillance in selected patients. In contrast, MD-IPMNs exhibit a substantially higher malignant potential (57–92%) and are more likely to harbor HGD or IC, for which surgical resection is typically recommended. Mixed-type IPMNs should be treated according to the same principles as MD-IPMNs [4,5,6].

Owing to this wide spectrum of biological behavior, IPMN represents a heterogeneous disease entity with considerable variability in clinical presentation, radiological findings, and oncologic potential. Accurate classification of IPMN subtype is therefore essential for appropriate risk stratification and treatment planning. Diagnostic imaging plays a central role in this process, as morphological classification relies primarily on imaging findings, including the degree of main pancreatic duct dilatation, the size and morphology of the cystic lesion, and associated features such as mural nodules or solid components.

Furthermore, diagnostic imaging is indispensable for longitudinal assessment, as changes in morphological features over time may indicate an increased risk of malignancy. Given the diversity of IPMN characteristics and their dynamic nature, high-quality and reproducible imaging is crucial for both accurate diagnosis and the formulation of an optimal treatment strategy. As such, the role of diagnostic imaging is fundamental and cannot be overlooked in the comprehensive management of patients with IPMN.

Overall, the prognosis of patients with IPMN-derived invasive carcinoma following surgical resection is more favorable than that of patients with conventional pancreatic ductal adenocarcinoma (PDAC) [7,8,9]. Despite this relatively better prognosis, IPMNs frequently exhibit multifocality, and recurrence of IPMN-derived carcinoma can develop in the remnant pancreas after surgery. In addition, both synchronous and metachronous occurrences of PDAC have been reported in patients with IPMN, along with the coexistence of other pancreatic neoplasms, including neuroendocrine tumors [10,11,12]. A review investigating the recurrence rate of non-invasive IPMN reported a median recurrence rate of 8.8% [13]. Several studies have examined the recurrence rate of invasive IPMN, reporting rates of 40–60% [14,15,16]. Risk factors for recurrence have also been identified, including main pancreatic duct dilatation (≥10 mm), a family history of pancreatic cancer, high-grade dysplasia (HGD) or invasive carcinoma (IC) in the primary lesion, positive surgical margins, and multifocal disease [17,18,19,20]. Importantly, disease recurrence may arise more than five years after the initial surgical intervention. Some patients experience recurrence more than 10 years after surgery, highlighting the necessity for prolonged and careful postoperative surveillance [17,21].

Although several international and regional clinical guidelines have been proposed for the management of IPMN, there remains considerable inconsistency regarding recommendations for the optimal duration and imaging modality of postoperative follow-up. As mentioned above, imaging findings play a central role in the preoperative evaluation of IPMN. However, the appropriate length of surveillance and the most effective imaging strategies after surgical resection have not been clearly established. Therefore, the present study aimed to retrospectively review postoperative imaging findings in patients who underwent resection for IPMN and to evaluate the optimal duration and modality of surveillance to improve long-term patient outcomes.

## 2. Materials and Methods

### 2.1. Patients

This retrospective study included 191 patients who underwent surgery for a preoperative diagnosis of IPMN at a single institution between January 2006 and May 2024, and had pathologically confirmed IPMN. During the study period, 1300 outpatients were clinically diagnosed with IPMN at the institution, of whom 201 patients were selected for surgical treatment based on clinical, radiological, and/or pathological indications. Among the 201 surgically treated cases, 10 patients were excluded from the final analysis because postoperative pathological evaluation revealed diagnoses other than IPMN. These excluded cases consisted of three patients with serous cystic neoplasms (SCN), two with benign pancreatic cysts, two with mucinous cystic neoplasms (MCN), two with PDAC, and one with a pancreatic neuroendocrine tumor. After applying these exclusion criteria, a total of 191 cases remained and were confirmed as IPMN on histopathological examination. Each lesion was classified based on the highest histological grade as LGD, HGD, and IC. One patient underwent two surgeries for IPMN at different locations during the study period, and each lesion was counted as a separate case.

### 2.2. Imaging Examinations

The hepatobiliary and pancreatic surgeons who performed the surgery were responsible for the follow-up of these patients. Medical images acquired at the surgical institution from the postoperative period until July 2025 were reviewed. CT, MRI, and ^18^F-fluorodeoxyglucose positron emission tomography/computed tomography (^18^F-FDG-PET/CT) scans interpreted and reported by the radiology department were included.

CT and MRI examinations included both plain and contrast-enhanced scans. For contrast-enhanced CT, nonionic contrast agents were administered using an injector. For single-phase imaging, a formulation with an iodine concentration of 300 mg/mL was injected at 2 mL/kg over 50 s, with venous-phase imaging performed 90 s after injection. For multiphase upper abdominal imaging, a formulation with an iodine concentration of 350 mg/mL was injected at 1.8 mL/kg over 30 s. The region of interest was the abdominal aorta at the level of the celiac artery. After a 100 H.U. increase in the CT number, arterial-phase imaging was performed 15 s later, portal venous phase imaging after 45 s, and delayed-phase imaging after 165 s. MRI was performed at field strengths of 1.5T or 3.0T. T2-weighted images, T1-weighted and fat-suppressed images (including in-phase and opposed-phase), diffusion-weighted images, and magnetic resonance cholangiopancreatography (MRCP) were acquired. For multiphase imaging of the upper abdominal region, 0.2 mL/kg of gadolinium contrast agent was administered at a rate of 3 mL/sec using an injector. The region of interest was the abdominal aorta, and the arterial phase was imaged using the bolus-tracking method. The portal venous phase was imaged 80 s later, with delayed-phase imaging done 180 s later. ^18^F-FDG-PET/CT scans were performed from the head to the thigh 60 min after administration of 3.7 MBq/kg of 18F-FDG. Images transferred from other hospitals were excluded. Examinations not including the upper abdomen were excluded, even if part of the surgical site was within the imaging range (e.g., chest CT).

### 2.3. Survey Methodology

Image interpretation reports generated by board-certified diagnostic radiologists were retrospectively reviewed to assess postoperative recurrence. Specifically, the time to detection of either intrapancreatic or extrapancreatic recurrence was calculated as the interval between the date of surgical resection and the date of the imaging examination at which recurrence was first identified. In addition, the imaging modality used for recurrence detection was recorded and categorized as non-contrast-enhanced or contrast-enhanced CT, non-contrast-enhanced or contrast-enhanced MRI, or ^18^F-FDG-PET/CT.

Intrapancreatic recurrence was defined radiologically as the appearance of a newly detected solid mass within the pancreatic remnant or at the pancreatic stump following surgery. Extrapancreatic recurrence was defined as the presence of disease outside the pancreas, including lymph node metastasis, invasion of the peripancreatic nerve plexus, peritoneal dissemination, or metastatic lesions in distant organs.

### 2.4. Statistical Analysis

Statistical analyses were performed using SPSS Statistics (version 24; IBM Corp., Armonk, NY, USA) and R (version 4.5.1; R Foundation for Statistical Computing, Vienna, Austria). Fisher’s exact test was used for categorical variables, and the Mann–Whitney U test was used for continuous variables. Statistical significance was set at *p* < 0.05.

## 3. Results

Among the 191 patients, 126 were men and 65 were women. The mean age was 68.8 years (range, 42–88 years, standard deviation (SD), 9.6 years). Lesions were in the pancreatic head in 134 patients, the body in 29 patients, and the tail in 28 patients. Pathological diagnoses included LGD in 112 patients, HGD in 49 patients, and IC in 30 patients.

Sixteen patients (8.3%) developed recurrence during the observation period. Seven patients experienced intrapancreatic recurrence, and ten patients experienced extrapancreatic recurrence. One patient developed a solid mass in the pancreatic head after lung metastasis was identified and was included in both categories.

### 3.1. Intrapancreatic Recurrence

Among the seven patients with intrapancreatic recurrence, four were men and three were women (*p* = 0.691). Preoperative IPMN was in the pancreatic head in three cases, the body in three cases, and the tail in one case (*p* = 0.083). Pathological diagnoses included LGD in two cases, HGD in one case, and IC in five cases; invasive carcinoma was significantly associated with intrapancreatic recurrence (*p* = 0.013). (Table 1).

**Figure 1:**
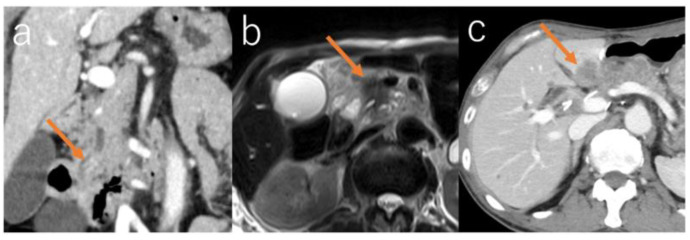
Examples of cases of intrapancreatic recurrence. (**a**) Case 3. Nodules in the IPMN lumen appeared 118 months after resection of the LGD in the pancreatic body. (**b**) Case 5. Solid mass in the pancreatic head which appeared 64 months after resection of IC in the pancreatic body. (**c**) Case 6. Solid mass from the anastomosis to liver which appeared 53 months after resection of HGD in the pancreatic head.

The mean time to detection of intrapancreatic lesions was 63.9 months (range, 16–118 months; median, 47 months; SD, 42.2 months). Five cases were detected using contrast-enhanced CT, one using non-contrast-enhanced MRI, and one using ^18^F-FDG-PET/CT. (Figure 1).

### 3.2. Extrapancreatic Recurrence

Among the 10 patients, eight were men and two were women (*p* = 0.499). Preoperative IPMN was located in the pancreatic head in five cases, the body in three cases, and the tail in two cases (*p* = 0.221). Pathological diagnoses included LGD in one case, HGD in one case, and IC in eight cases. Extrapancreatic recurrence (metastasis or dissemination) was more common in the IC group (*p* < 0.001). (Table 2).

**Figure 2:**
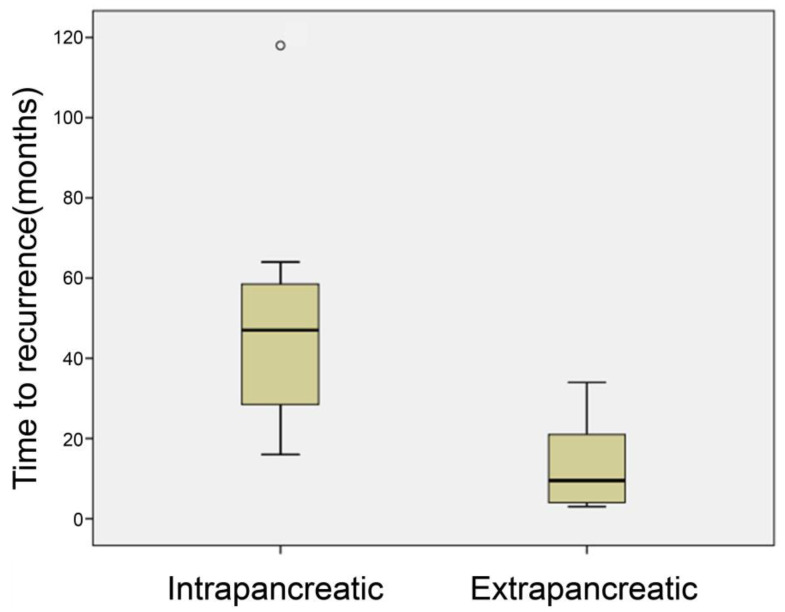
Time to intrapancreatic/extrapancreatic recurrence.

**Figure 3:**
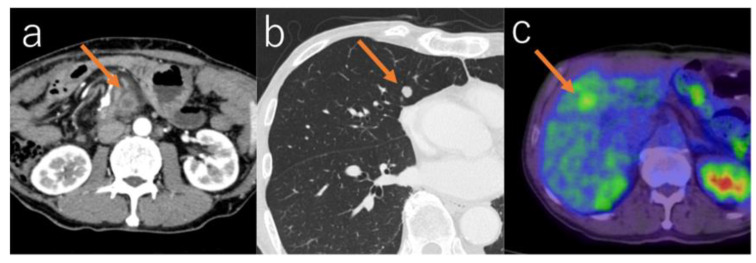
Examples of cases of extrapancreatic recurrence. (**a**) Case 2. Lymph node metastasis which appeared 10 months after resection of HGD in the pancreatic body. (**b**) Case 5. Pulmonary metastasis which appeared 23 months after resection of IC in the pancreatic head. (**c**) Case 8. Liver metastasis which appeared 4 months after resection of IC in the pancreatic head.

The mean time to detection of extrapancreatic lesions was 12.0 months (range, 3–34 months; median, 9.5 months; SD, 9.5 months), which was significantly shorter than that for intrapancreatic lesions (*p* = 0.005) (Figure 2). Eight cases were detected using contrast-enhanced CT, one using non-contrast-enhanced CT, and one using ^18^F-FDG-PET/CT. (Figure 3).

## 4. Discussion

Several clinical guidelines advocate postoperative surveillance following surgical resection of IPMN. The European evidence-based guidelines for pancreatic cystic neoplasms, as well as the clinical guidelines issued by the American College of Gastroenterology, recommend that patients with IC arising from IPMN undergo postoperative surveillance in accordance with established protocols for PDAC. For patients who undergo resection for noninvasive IPMN, including LGD and HGD, these guidelines recommend follow-up imaging at intervals of 6 months to 1 year, depending on the estimated risk of recurrence. MRI and endoscopic ultrasound (EUS) are commonly employed imaging modalities in this setting [22,23]. Similarly, the international clinical practice guideline, also known as the Kyoto guideline, recommends postoperative surveillance at intervals ranging from 6 months to 1 year, although it does not specify a preferred imaging modality [24].

In the present study, contrast-enhanced CT demonstrated a high detection rate for postoperative recurrence, identifying five of seven intrapancreatic recurrences and eight of ten extrapancreatic recurrences. Overall, recurrence was observed in 8.3% of the study population. Although the majority of recurrent cases occurred following surgical resection for IC, contrast-enhanced CT was also capable of detecting recurrences in patients who had undergone resection for LGD and HGD lesions. These results suggest that contrast-enhanced CT may represent a useful and reliable modality for postoperative surveillance in patients with IPMN. Notably, international guidelines already reference contrast-enhanced CT as an important tool for preoperative evaluation. Previous studies have identified contrast-enhancing mural nodules and enlarged lymph nodes as risk factors for postoperative recurrence [25], and it has been reported that most recurrent lesions can be detected using contrast-enhanced CT with moderate to high interobserver agreement [26]. To our knowledge, this study is the first to directly compare the number of recurrent lesions detected by imaging specialists using contrast-enhanced CT with those detected by other imaging modalities.

Among the intrapancreatic recurrences observed in this study, only one case (case 3) demonstrated a cystic background, whereas the remaining lesions appeared as solid masses without associated cystic components. Although imaging modalities and follow-up protocols were not standardized across patients, contrast-enhanced CT proved particularly useful for postoperative surveillance because most recurrent lesions manifested as solid tumors rather than cystic changes.

In addition, one intrapancreatic recurrence and one extrapancreatic recurrence were detected using ^18^F-FDG-PET/CT. ^18^F-FDG-PET/CT has been reported to be useful in the preoperative setting for differentiating benign from malignant IPMN [27], and parameters such as the tumor-to-liver uptake ratio have been suggested as helpful diagnostic indicators [28]. However, other studies have reported no significant advantage of ^18^F-FDG-PET/CT over conventional imaging modalities [29]. To date, there have been no published reports specifically evaluating the utility of ^18^F-FDG-PET/CT for postoperative surveillance following IPMN resection, and further investigation is therefore warranted.

In the present analysis, the time to detection of intrapancreatic recurrence was significantly longer than that of extrapancreatic recurrence. Three potential mechanisms of intrapancreatic recurrence have been proposed: (1) recurrence at the surgical margin, (2) intrapancreatic metastasis, and (3) the development of multiple independent lesions within the pancreas [30]. The prolonged time to detection observed in intrapancreatic recurrence in this study may be attributable to the development of de novo lesions independent of the original IPMN. Previous studies have identified preoperative histological diagnosis of IC as a significant risk factor for intrapancreatic recurrence [17,31], a finding that is consistent with the results of our study. Among patients with extrapancreatic recurrence, one patient had undergone resection for LGD; however, concomitant pancreatic cancer was resected simultaneously, suggesting that the recurrence may have represented metastasis from the coexisting pancreatic cancer rather than progression of the IPMN itself.

The shortest interval to detection of intrapancreatic recurrence was 16 months, whereas the shortest interval to detection of extrapancreatic recurrence was slightly longer than 3 months. Based on these findings, postoperative surveillance for patients at low risk of extrapancreatic recurrence may be appropriately performed at approximately annual intervals. In contrast, for patients considered to be at high risk, more intensive surveillance at intervals of approximately every 3 to 4 months may be warranted. There remains a risk of developing high-grade or invasive IPMN and PDAC in pancreatic regions other than the resected site; therefore, lifelong surveillance is considered necessary for these patients.

Although the number of studies evaluating long-term outcomes after IPMN surgery has increased in recent years, there remains substantial heterogeneity in the definition of recurrence or disease progression across studies. Some reports focus primarily on the development of high-grade or invasive IPMN and PDAC, which typically require surgical intervention [18,32], whereas others include postoperative cyst growth or progression of main pancreatic duct dilatation during surveillance as indicators of disease progression [33,34]. In patients with IPMN, surgical intervention should be considered, particularly when the main pancreatic duct is dilated to 5 mm or greater, as this finding may indicate malignancy [35]. In addition, cysts with a diameter of 30 mm or larger, as well as cysts demonstrating rapid growth—defined as an increase of 2 mm or more per year or 10 mm or more during follow-up—should also be regarded as potentially malignant [36,37]. These characteristics are classified as high-risk stigmata (HRS) and worrisome features (WF), both of which are associated with an increased risk of malignant transformation in IPMN [24]. The recommended follow-up intervals, diagnostic modalities, and indications for surgery differ according to the presence or absence of these features. Main pancreatic duct dilatation of 10 mm or greater is classified as HRS, while dilatation of 5–9 mm is considered a WF. In addition, cysts measuring greater than 30 mm in diameter or exhibiting a growth rate of at least 2.5 mm per year are also classified as WF. Although these features are important for the diagnosis and management of IPMN, interpretation can be challenging in the postoperative setting. Main pancreatic duct dilatation may result from early postoperative edema or postoperative scarring, and anatomical changes following pancreaticoduodenectomy are particularly difficult to assess. Moreover, cystic lesions can be difficult to distinguish from retention cysts or dilated branch pancreatic ducts. During the study period, some patients who were preoperatively diagnosed with IPMN were ultimately found to have benign cysts on pathological examination. For these reasons, the present study focused primarily on solid lesions, which are more clearly defined and represent more appropriate candidates for intervention.

Several limitations of this study should be acknowledged. First, due to its retrospective design, imaging intervals, imaging modalities, and imaging protocols were not standardized across the study population. Second, in some cases, recurrent lesions were not surgically resected, and therefore detailed histopathological confirmation was unavailable. Furthermore, because it is often difficult to definitively distinguish true postoperative recurrence from newly developed pancreatic cancer, all detected lesions were classified as “recurrences” in this study. Finally, given the relatively small number of recurrent cases, further accumulation of data is required, particularly to clarify recurrence patterns following resection of LGD and HGD lesions.

## 5. Conclusions

Extrapancreatic lesions appeared sooner after IPMN surgery than intrapancreatic lesions. Contrast-enhanced CT was frequently used to detect recurrent lesions, suggesting its potential utility in postoperative surveillance. For patients at low risk of intrapancreatic recurrence, surveillance at approximately one-year intervals was considered appropriate, whereas for high-risk patients, surveillance every 3–4 months was considered appropriate.

## Figures and Tables

**Table 1:** Cases with intrapancreatic recurrence.

Case No.	Sex	Age	Lesion	Pathologic Diagnosis	Time to Recurrence (Month)	Modality
1	F	78	Head	IC	47	Contrast-enhanced CT (solid mass in the pancreatic tail)
2	F	77	Body	IC	16	Contrast-enhanced CT (solid mass in the pancreatic head, left retroperitoneal mass)
3	F	57	Body	LGD	118	Contrast-enhanced CT (nodule in the IPMN lumen of the pancreatic uncinate)
4	M	75	Head	IC	17	^18^F-FDG-PET/CT (solid mass in the pancreatic tail)
*5	M	82	Body	IC	64	Non-contrast-enhanced MRI (solid mass in the pancreatic head)
6	M	66	Head	HGD	53	Contrast-enhanced CT (solid mass from the anastomosis to liver)
7	M	74	Tail	LGD	40	Contrast-enhanced CT (solid mass inside the pancreatic uncinate)

* Cases 5 and 6 with intrapancreatic and extrapancreatic recurrences, respectively, are the same.

**Table 2:** Cases with extrapancreatic recurrence.

Case No.	Sex	Age	Lesion	Pathological Diagnosis	Time to Recurrence (month)	Modality
1	F	66	Head	IC	9	Contrast-enhanced CT (solid mass around abdominal aorta and ceriac artery (CeA), liver metastasis)
2	M	63	Body	HGD	10	Contrast-enhanced CT (solid mass around superial mesenteric artery/vein (SMA/V))
3	M	78	Head	IC	4	Non-contrast-enhanced CT (liver metastasis, peritoneal dissemination)
4	F	79	Head	IC	3	Contrast-enhanced CT (solid mass around CeA, SMA and abdominal aorta, cervical and thoracic metastases)
5	M	80	Head	IC	23	Contrast-enhanced CT (pulmonary metastases)
*6	M	82	Body	IC	34	Contrast-enhanced CT (pulmonary metastases)
7	M	82	Tail	IC	14	Contrast-enhanced CT (pulmonary metastases)
8	M	69	Head	IC	4	^18^F-FDG-PET/CT (liver metastases)
⁑9	M	70	Body	LGD	21	Contrast-enhanced CT (pulmonary metastases)
10	M	80	Tail	IC	7	Contrast-enhanced CT (liver metastases)

* Cases 5 and 6 of intrapancreatic and extrapancreatic lesions, respectively, were the same patient. ⁑ Case 9 with an extrapancreatic lesion underwent simultaneous resection of LGD IPMN and coexisting pancreatic cancer.

## Data Availability

The data presented in this study are available on request from the corresponding author due to the patients’ privacy.
